# Contribution of Val/Ile87 residue in the extracellular domain in agonist-induced current responses of the human and rat P2X7 receptors

**DOI:** 10.1007/s11302-020-09730-1

**Published:** 2020-10-08

**Authors:** Emily A Caseley, Stephen P Muench, Lin-Hua Jiang

**Affiliations:** 1 Leeds Institute of Rheumatic and Musculoskeletal Medicine, St James’s University Hospital, Faculty of Medicine and Health, Leeds, UK; 2 School of Biomedical Sciences, Faculty of Biological Sciences, University of Leeds, Leeds, LS2 JT UK

**Keywords:** P2X7 receptor, Species difference, Agonist-induced currents, Whole-cell recording

## Abstract

The P2X7 receptor (P2X7R) is an ATP-gated cation channel with a critical role in many physiological and pathological processes, and shows prominent functional differences across mammalian species, exemplified by larger current responses of the rat (r) P2X7R to ATP and its analogue BzATP and a greater sensitivity to agonists compared with the human (h) P2X7R. Here, we showed that substitution of Val87 residue in the extracellular domain of the hP2X7R with isoleucine in the rP2X7R increased the current responses of the hP2X7R to both ATP and BzATP. Conversely, introduction of reciprocal I87V mutation in the rP2X7R led to a noticeable but statistically insignificant reduction in the current responses of the rP2X7R to ATP and BzATP. The mutations did not affect the sensitivity of the human and rat P2X7Rs to ATP and BzATP. These results suggest a contribution of Val/Ile87 in agonist-induced current responses of human and rat P2X7Rs, which helps to better understand the molecular determinants for species-dependent function of the mammalian P2X7Rs.

**Electronic supplementary material:**

The online version of this article (10.1007/s11302-020-09730-1) contains supplementary material, which is available to authorized users.

## Introduction

P2X receptors (P2XRs) comprise a group of seven trimeric, nonselective cation channels that are selectively activated by extracellular ATP [1]. The mammalian P2X7 receptor (P2X7R) is a key mediator of responses induced by high levels of ATP associated with tissue inflammation and damage, hypoxia, and emotional stress, and plays a critical role in diverse pathologies such as inflammatory disorders, neurodegenerative diseases, mood disorders, and cancers [2–6]. The P2X7Rs of different species exhibit striking differences in functional properties; it has been well documented that the agonist concentrations evoking half of the maximal response (EC_50_) for ATP and its analogue BzATP are several-fold higher at the human (h) P2X7R than at the rat (r) P2X7R and, additionally, agonist-evoked responses from the hP2X7R are smaller than those from the rP2X7R [7–10]. Previous studies identified two residues, one at position 155 in the extracellular domain and the other at 348 in the second transmembrane domain, as important determinants for the difference in agonist-induced current responses between human and rat P2X7Rs [11, 12]. In this short communication, we report residue at position 87 in the extracellular domain that contributes the current responses of the human and rat P2X7Rs to ATP and BzATP.

## Methods

### Homology modelling and ligand docking

The structures of the rP2X7R in the closed and ATP-bound open states (Protein Data Bank code 6U9V and 6U9W, respectively) were used to produce the structural models of the hP2X7R. Modeller version 9.12 [13] was used to produce these models, which were analysed using MolProbity [14] as detailed in our previous studies [15, 16]. AutoDock version 4.2 [17] was used to dock ATP and BzATP to a cavity file consisting of a 15-Å sphere surrounding the P2X7R ATP-binding site and affinity grid files were generated using the auxiliary program AutoGrid.

### Site-directed mutagenesis

The cDNA for wild-type (WT) human or rat P2X7 receptor subunit with an EE epitope in the C-terminus was subcloned in pcDNA3.1 vector and used in our previous studies [11, 15]. Point mutations were introduced using a PCR-based protocol we previously described [18] (for more details, see supplemental information) and confirmed by commercial sequencing (Beckman Coulter Genomics).

### Expression of P2X7R and patch-clamp recording

WT and mutant P2X7Rs were transiently expressed in human embryonic kidney (HEK) 293 cells, and agonist-induced whole-cell currents were recorded using patch-clamp technique, as previously described [11, 15]. The EC_50_ values were derived by fitting the concentration-response relationship curves to the Hill equation [14]. Further details are presented in supplemental information. Agonist-induced currents recorded in parallel experiments were used for comparisons.

### Data analysis

All results, where appropriate, are presented as the mean ± standard error of the mean (SEM).

Statistical analysis was carried out using Student’s *t* test for two groups or one-way analysis of variance test and Tukey’s post hoc test for more than two groups, with the difference considered to be significant at *p* < 0.05.

## Results

### Effects of mutating residues in the extracellular domain in hP2X7R on BzATP-induced currents

**Fig. 1:**
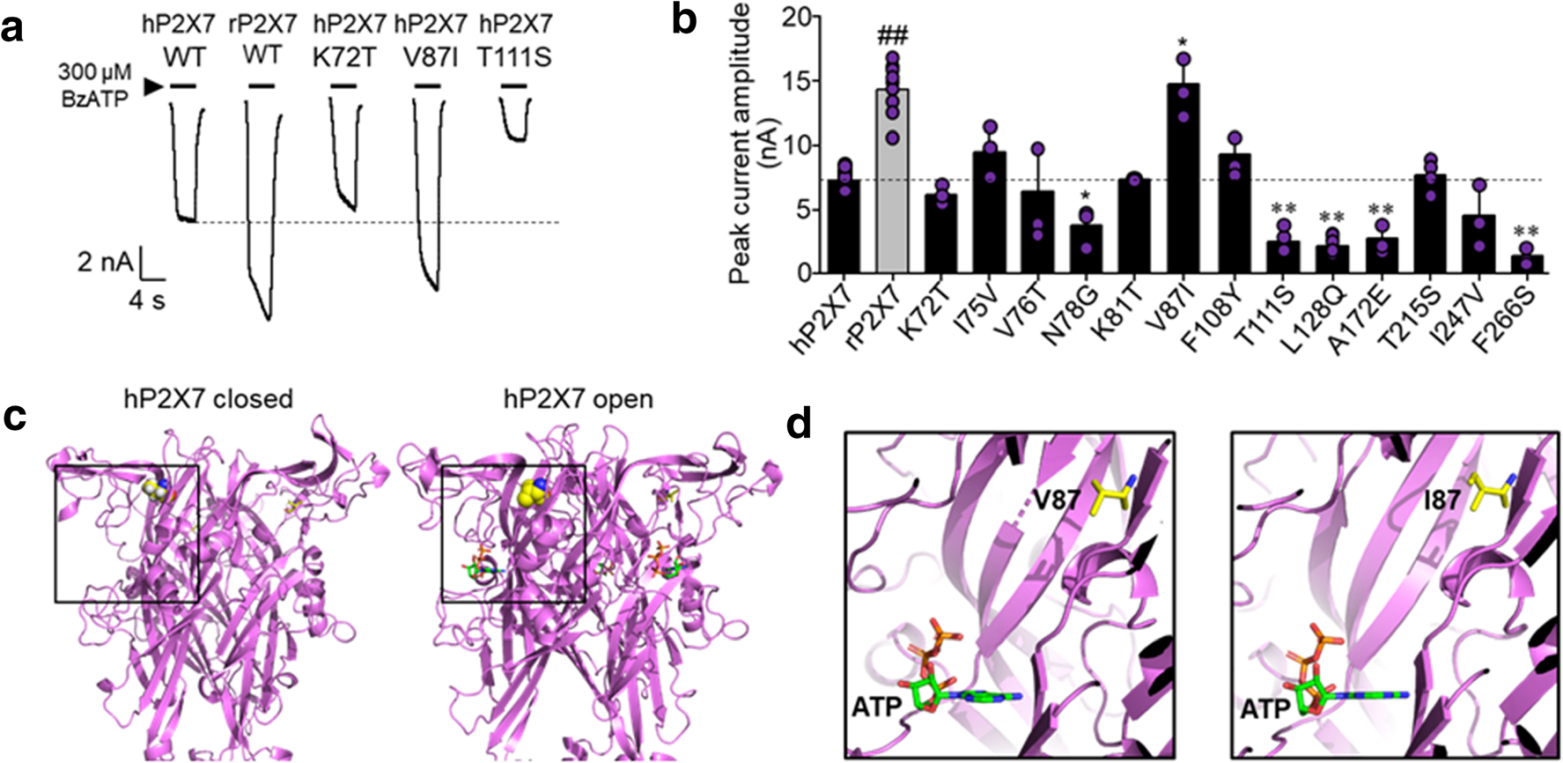
Effects of mutating residues in the extracellular domain that differ between human and rat P2X7Rs on BzATP-induced hP2X7R-mediated currents. **a** Representative currents evoked by 300 μM BzATP in HEK293 cells expressing wild-type (WT) human (h) P2X7R, WT rat (r) P2X7R, or mutant hP2X7R as indicated. **b** Summary of mean BzATP-induced current amplitude for indicated P2X7Rs. *n* = 3–4 cells for each case, 8–9 cells for hP2X7 and rP2X7 controls. *p < 0.05 and **p < 0.01 compared with WT hP2X7R. ^##^*p* < 0.01 indicating difference between human and rat WT P2X7Rs. **c** Structural models of the hP2X7R showing the location of Val87, represented as spheres, in closed state (left) and ATP-bound open state (right). **d** Zoomed-in view of Val87 (V87) in the hP2X7R (left) and Ile87 (I87) in the rP2X7R (right), relative to the ATP-binding site

Sequence analysis of the extracellular domain of the human (and monkey) versus the rat (and mouse) P2X7Rs [7, 19, 20] identified 25 residues of interest (Fig. 1S). We chose a subset of 13 residues and substituted each of them in the hP2X7R with that in the rP2X7R, expressed the mutant hP2X7Rs in HEK293 cells in parallel with the WT human and rat P2X7Rs, and assessed the mutational effects on the current responses to 300 μM BzATP, a maximal concentration (Fig. 1a, b). As anticipated, BzATP elicited much smaller currents from WT-hP2X7R than WT rP2X7R (Fig. 1b). Seven mutations had no effect on, and five mutations reduced, BzATP-induced currents (Fig. 1b). V87I was the only mutation increasing BzATP-induced currents, with the mean amplitude significantly higher than that from WT hP2X7R and comparable with that from WT rP2X7R (Fig. 1b). Val/Ile87 is located in the top part of the extracellular domain of the receptors (Fig. 1c, d).

### Effects of reciprocally mutating residues at position 87 in human and rat P2X7Rs on agonist-evoked currents

**Fig. 2:**
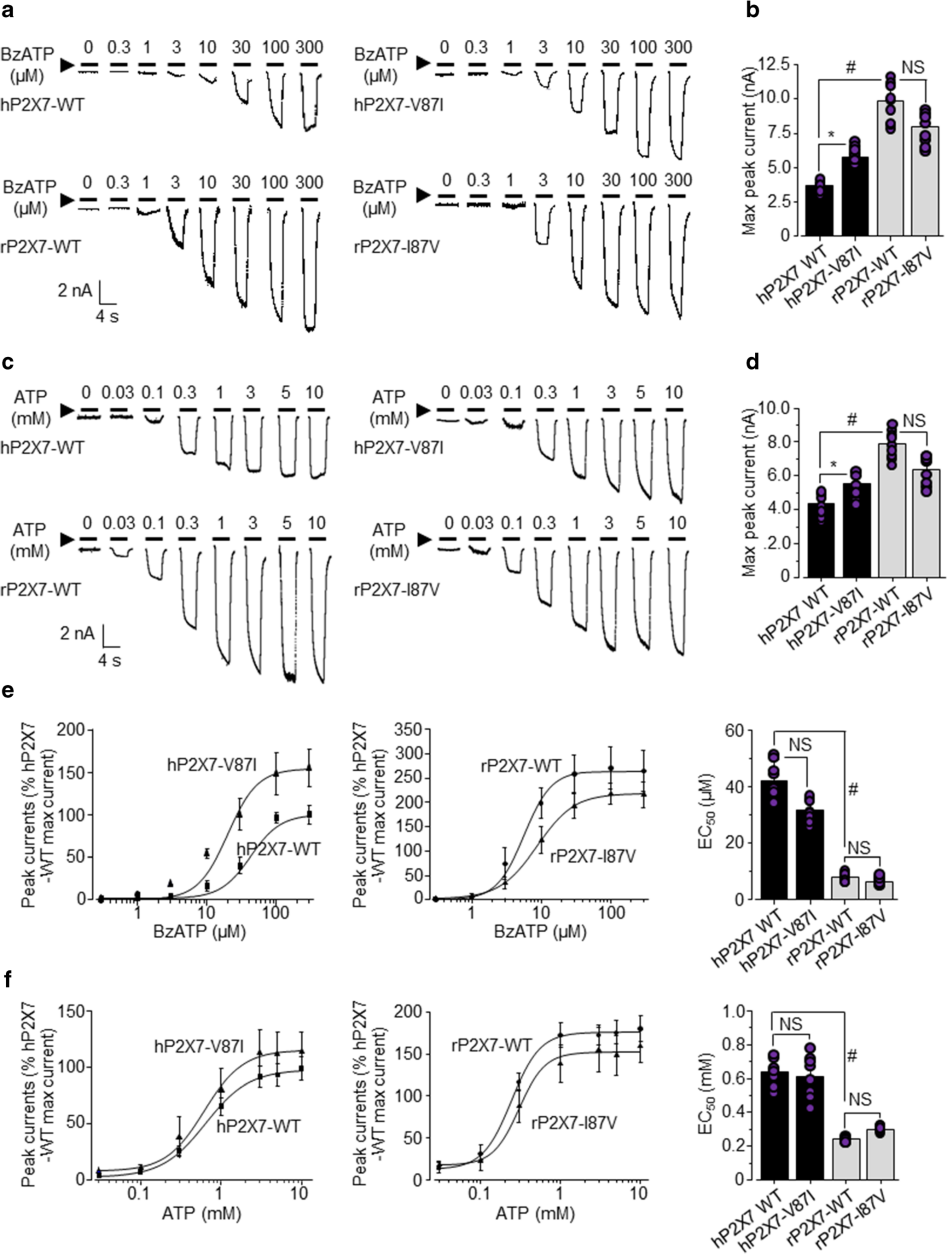
Effects of reciprocally mutating Val/Ile87 on agonist-induced currents mediated by human and rat P2X7Rs. **a** Representative current responses to BzATP at indicated concentrations from HEK293 cells expressing wild-type (WT) or mutant human (h) or rat (r) P2X7R as indicated. **b** Summary of the mean current amplitudes evoked by 300 μM BzATP from cells expressing WT or mutant P2X7R. **c** Representative current responses to ATP at indicated concentrations from cells expressing WT or mutant P2X7R as indicated. **d** Summary of the mean current amplitudes evoked from cells expressing WT or mutant P2X7R by 10 mM ATP. **e**, **f** Summary of the mean concentration-current response relationship curves and the EC_50_ values of BzATP (**e**) and ATP (**f**) for WT and mutant P2X7Rs. The solid lines (**e**, **f**) represent the best fits of the mean data to Hill equation. *p < 0.05 compared with WT hP2X7R; ^#^*p* < 0.05 between human and rat WT P2X7Rs; NS, no significant difference

To better understand the contribution of Val87 in the hP2X7R and Ile87 in the rP2X7R in species difference, we next examined the effect of V87I mutation on the hP2X7R and the effect of reciprocal mutation I87V on the rP2X7R in their current responses to a range of BzATP concentrations (Fig. 2a, b). BzATP at all concentrations elicited larger currents from hP2X7R-V87I than WT hP2X7R (top two panels in Fig. 2a). The mean maximal current amplitude from hP2X7R-V87I was significantly larger than that from WT hP2X7R (Fig. 2b). Conversely, BzATP at all concentrations evoked smaller currents from rP2X7R-I87V relative to WT rP2X7R (bottom two panels in Fig. 2a). The mean maximal current amplitude from rP2X7R-I87V was smaller than that from WT rP2X7R, but the difference did not reach statistical significance (Fig. 2b). We also examined the effects of both mutations on the current responses of human and rat P2X7Rs to a range of ATP concentrations (Fig. 2c, d). Similarly, ATP at all concentrations evoked larger currents from hP2X7R-V87I than from WT hP2X7R, with the maximal current amplitude from hP2X7R-V87I significantly larger than that from WT hP2X7R (Fig. 2c, d). Introduction of the I87V mutation in the rP2X7R decreased the current responses to ATP at all concentrations. The maximal current amplitude from rP2X7R-I87V was smaller than that from WT rP2X7R and again the reduction was not statistically significant (Fig. 2d). Collectively, these results indicate that reciprocal mutation of the residue at position 87 interchanged the current responses of the human and rat P2X7Rs to both BzATP and ATP, albeit with the human-to-rat mutation resulting in more prominent or significant effects.

It is known that the hP2X7R is less sensitive to agonists than the rP2X7R [7–11]. We further examined the effects of reciprocally mutating the residue at position 87 on the sensitivity of human and rat P2X7Rs to BzATP and ATP by determining the EC_50_ values. The BzATP EC_50_ for WT hP2X7R was approximately 5-fold greater than that for WT rP2X7R (Fig. 2e) and the ATP EC_50_ for WT hP2X7R was 3-folder higher than that for WT rP2X7R (Fig. 2f). The BzATP EC_50_ for hP2X7R-V87I was similar to that for WT hP2X7R, and the BzATP EC_50_ for rP2X7R-I87V was close to that for WT P2X7R (Fig. 2e). Similarly, the ATP EC_50_ for both hP2X7R-V87I and rP2X7R-I87V showed no difference to that for respective WT receptors (Fig. 2f). These results indicate no effect of the mutations on the sensitivity of the human and rat P2X7Rs to both BzATP and ATP.

## Discussion

The mammalian P2X7Rs are well known for their striking inter-species functional differences, as shown by the agonist-induced current responses and agonist sensitivity [7, 10, 11]. To gain a better understanding of the molecular determinants for such species difference between the human and rat P2X7Rs, we selected 13 residues in the extracellular domains and examined the effects of substituting individual residue in the hP2X7R with that in the rP2X7R on the current responses to BzATP at a maximal concentration (Fig. 1a, b). All the mutations, except for V87I, led to either no change or a reduction in BzATP-induced current response. Here, we did not further examine these mutants and how these mutations induced loss of function mechanistically remains unclear. V87I increased BzATP-induced hP2X7R-mediated currents, with the current amplitude close to that from WT rP2X7R (Fig. 1b). We further examined this gain-of-function mutation in the hP2X7R and the reciprocal I87V mutation in the rP2X7R in terms of their effects on the agonist-induced current amplitude and the agonist sensitivity at the human and rat P2X7Rs. We confirmed the reported differences in the current amplitude and the sensitivity to BzATP and ATP between human and rat P2X7Rs (Fig. 2a–d). Importantly, our results showed that the current responses from V87I-hP2X7R to both BzATP (Fig. 2a, b) and ATP (Fig. 2c, d) were consistently higher than those from WT-hP2X7R. Conversely, introduction of the I87V mutation in the rP2X7R reduced the current responses to both BzATP (Fig. 2a, b) and ATP (Fig. 2c, d), though the differences were statistically insignificant. Moreover, our results showed no effect of V87I in the hP2X7R or I87V in the rP2X7R on the EC_50_ values for ATP and BzATP (Fig. 2e, f). Collectively, these results suggest that Val/Ile87 residue mainly influences the difference in agonist-induced current responses between human and rat P2X7Rs.

The recently determined rP2X7R structure [21] provides useful information to understand or rule out the mechanisms by which Val/Ile87 residue contributes to the mutational effects on human and rat P2X7Rs. This residue is positioned distant to the agonist-binding site in the human, rat, and mouse P2X7Rs (Fig. 1c, d and Fig. S2A) [22], and thus, the mutations are unlikely to influence agonist binding, which is consistent with no effect on the sensitivity to ATP or BzATP (Fig. 2e, f). As we previously showed, Ala/Thr348 in the second transmembrane domain lining the ion-conducting pore contributes to the difference in the current responses of human and rat P2X7Rs to ATP via affecting the ion conductance [11]. This possibility can be ruled out for Val/Ile87 because it is far away from the transmembrane pore. Our previous study suggests that His/Tyr155 in the extracellular domain differentially modulates the cell surface expression of human and rat P2X7Rs and therefore contributes to the difference in the current responses of human and rat P2X7Rs to ATP [11]. Immunofluorescent imaging revealed no noticeable mutational effect on the expression and subcellular distribution of human and rat P2X7Rs (Fig. S3). The location of Val/Ile87 is at the top of the ‘head’ region of the P2X7Rs, with Val87 in the hP2X7R in the vicinity of residues including Glu186 [23] and Gln187 [24] (Fig. S2B), mutation of which were shown to affect hP2X7R activation, prompting us to hypothesise that Val/Ile87 may impact the closure of the ATP-binding vestibule that induces the conformational changes essential for channel opening [25]. Further investigations are required to examine this hypothesis. Residues flanking Val87 in the hP2X7R were reported to participate in allosteric binding of AZ10606120, a human and rat P2X7R antagonist [26]. It would be interesting to examine whether Val/Ile87 contributes to the action of antagonists exhibiting species differences.

In summary, this study reports contribution of extracellular Val/Ile87 residue in agonist-induced current responses of human and rat P2X7Rs, which helps us better understand the molecular determinants for species difference in the function of the mammalian P2X7Rs.

## Electronic supplementary material

ESM 1 (PDF 794 kb)
