# Supplementary material for: Contribution of Val/Ile87 residue in the extracellular domain in agonist-induced current responses of the human and rat P2X7 receptors
Source: Purinergic Signal. 2020 Oct 8;16(4):485–90. doi: 10.1007/s11302-020-09730-1 (PMC7855165; doi:10.1007/s11302-020-09730-1)
Supplement: Supplementary file 1 — (PDF 794 kb) [file 11302_2020_9730_MOESM1_ESM.pdf]

Supplementary information

Methods

Site-directed mutagenesis
Plasmids encoding the wild type (WT) human or rat P2X7 receptor with an EE epitope in the C-terminus used this study were generated as described in our previous studies [11,15], and point mutations were introduced using a protocol we previously described in detail [17]. In brief, a 50 µL-PCR sample containing 300 nM of each primer, 200 µM dNTP mix, 100 ng cDNA and 2.5 U PfuUltra DNA polymerase (Agilent). PCR consisted of the following steps: 96°C for 60 s, 18 cycles consisting of 96°C for 50 s, 60°C for 50 s, 68°C for 14 min and a final step of 68°C for 30 min. Following treatment with DpnI (ThermoFisher Scientific) for 60 min at 37 °C, the resulting PCR product was transformed into competent *E. coli* cells (Strategene). Plasmids were extracted using a mini-DNA preparation kit (QIAGEN) and mutations confirmed by commercial sequencing (Beckman Coulter Genomics).

Cell culture and transient transfection
Human embryonic kidney (HEK) 293 cells were cultured in Dulbecco's Modified Eagle Medium (DMEM) supplemented with 10% foetal bovine serum (FBS) at 37°C and 5% CO_2, under humidified conditions. Cells were seeded in 6-well plates at 70-80% confluency prior to transfection and cells in each well were transfected using Lipofectamine2000 (Life Technologies) with 1 µg plasmid for the WT or mutant P2X7 receptor and 0.1 µg plasmid for enhanced green fluorescent protein, according to the manufacturer's instructions.

Whole-cell patch-clamp current recording
Patch-clamp recordings were performed using an Axopatch 200B amplifier (Molecular Devices) to record whole-cell currents at room temperature, and an RSC-160 rapid solution changer (Biologic Science Instruments) to apply ATP and BzATP. Cells were seeded onto 10-mm glass coverslips 20-24 hrs post transfection and recordings were taken from single, GFP-positive cells. Patch microelectrodes with a resistance of 1-5 MΩ were produced using borosilicate glass capillaries (World Precision Instruments) and cells were kept at a holding potential of -80 mV. Standard extracellular solution consisted of 147 mM NaCl, 2 mM KCl, 1 mM $MgCl_2$, 2 mM $CaCl_2$, 10 mM HEPES and 13 mM glucose, pH 7.3. Intracellular solution contained 145 mM NaCl, 10 mM EDTA and 10 mM HEPES, pH 7.3. Due to the inhibitory effect of divalent cations on the P2X7 receptors, agonist-induced currents were measured in low divalent extracellular solution consisting of 147 mM NaCl, 2 mM KCl, 0.3 mM $CaCl_2$, 10 mM HEPES and 22 mM glucose, pH 7.3. The whole-cell configuration was established in standard extracellular solution which was then replaced with low divalent extracellular solution. A low concentration of agonist was applied for 4 s at 2 min intervals until the current was fully facilitated and the current amplitude remain stable, before applying 300 μM BzATP in the initial screening of point mutants (Fig. 1), or applying increasing concentrations of agonist for 4 s at 2 min intervals to obtain concentration-current responses relationship (Fig. 2). The current recordings were analyzed with pClamp 10.3 software. The EC_{50} values for ATP and BzATP were derived by least-squares fitting of the concentration-current response relationship curve from individual cells to the Hill equation: $I = I_{max}/(1+ (EC_{50}/[A])^n)$, where I is the peak current evoked by given agonist concentrations ([A]), I_{max} is the maximal current, and n is the Hill coefficient. All currents were expressed as percentage of the average maximal currents recording from cells expressing the WT human P2X7 receptor in parallel experiments. Figures show the curves fitted to the mean data from all cells.

Immunofluorescent confocal imaging
Immunofluorescent confocal imaging was used to examine the cellular distribution of the P2X7 receptors expressed in HEK293 cells as described in our previous study [11]. In brief, HEK293 cells transfected as described above were seeded onto 13-mm cover slips, 20,000 cells per slip, and incubated at 37ºC overnight. Cells were rinsed with PBS before being

briefly incubated with a 50:50 mixture of PBS and Zamboni's fixative solution (15% (v/v) picric acid and 5.5% (v/v) formaldehyde in PBS), followed by 100 % Zamboni's fixative at room temperature for 1 hr. PBS was added for 5 min and then removed; this wash was repeated 3 times before the addition of blocking solution (10% (v/v) goat serum in PBST (0.4% (v/v) Triton X-100 dissolved in PBS)) for 1 hr at room temperature. Mouse anti-EE primary antibody was added into the blocking solution (1:1000 dilution) and the cells incubated at 4°C overnight. The cells were washed with PBS three times as described above, and then incubated in blocking solution containing goat anti-mouse IgG secondary antibody conjugated with fluorescein isothiocyanate at 1:5000 for 1 hr at room temperature. The cells were washed once in PBS and twice in water, and the cover slips were mounted onto microscope slides with SlowFadeGold Antifade mountant with DAPI (Invitrogen) and stored at 4°C. Images were captured using a Zeiss LSM 880 upright microscope and ZEN imaging software.

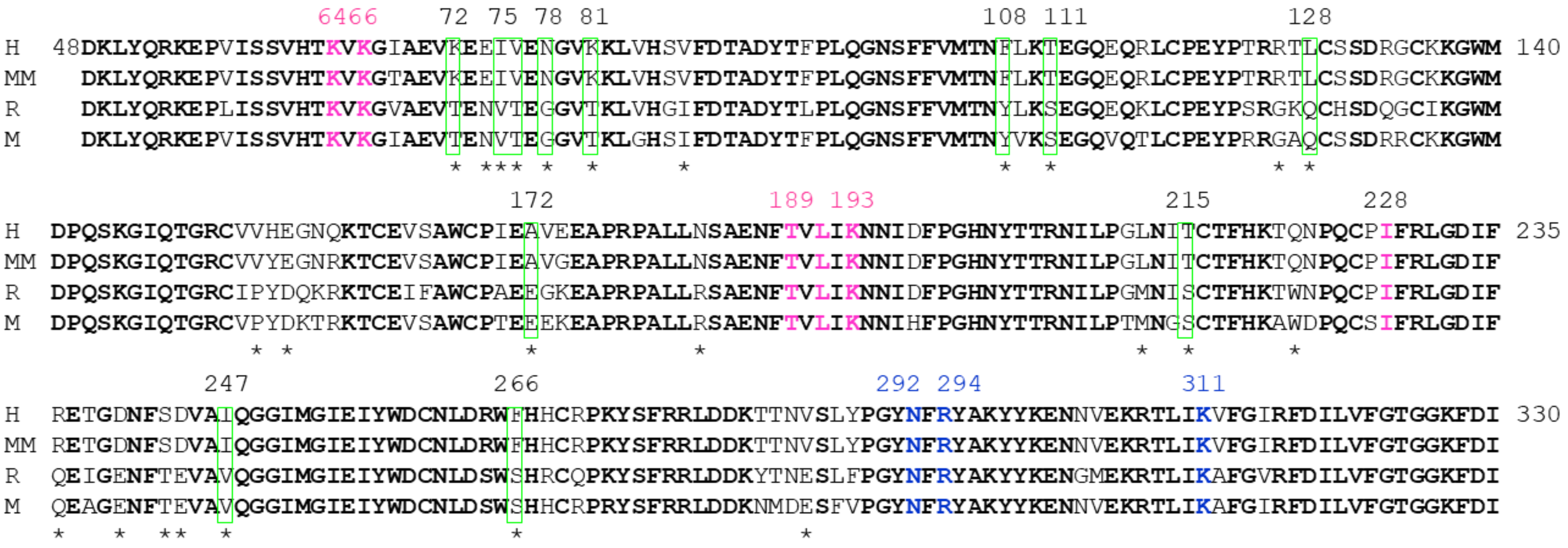

Figure S1 Sequence analysis of the extracellular domains of the P2X7 receptors

Amino acid residue Alignment of the extracellular domain of the human (H), macaque monkey (MM), rat (R) and mouse (M) P2X7 receptors. The conserved residues are in bold. The residues coordinating inter-subunit ATP binding from one subunit are denoted in cyan and from neighbouring subunit in blue. * indicates residues above that differ in the human and monkey receptors versus rat and mouse receptors, with residues examined in this study highlighted in green rectangle.

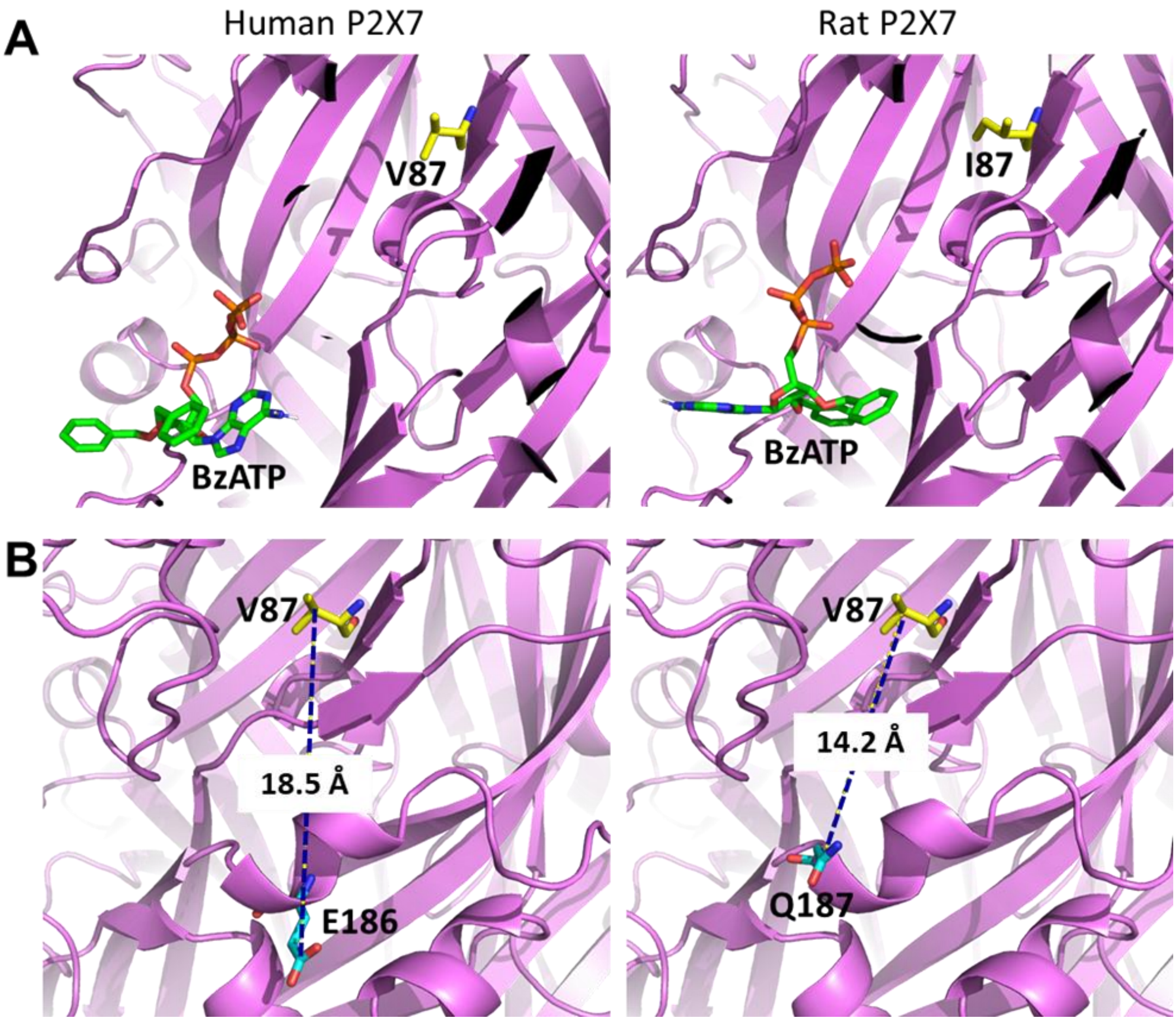

Figure S2 Structure or structural models of the rat and mouse P2X7 receptors
A. Predicted BzATP binding to the human (left) and rat (right) P2X7 receptors. **B**. Zoomed-in views of the relative proximity of Val87 to Glu186 (left) and Gln187 (right) in the human P2X7 receptor in ATP-bound open state.

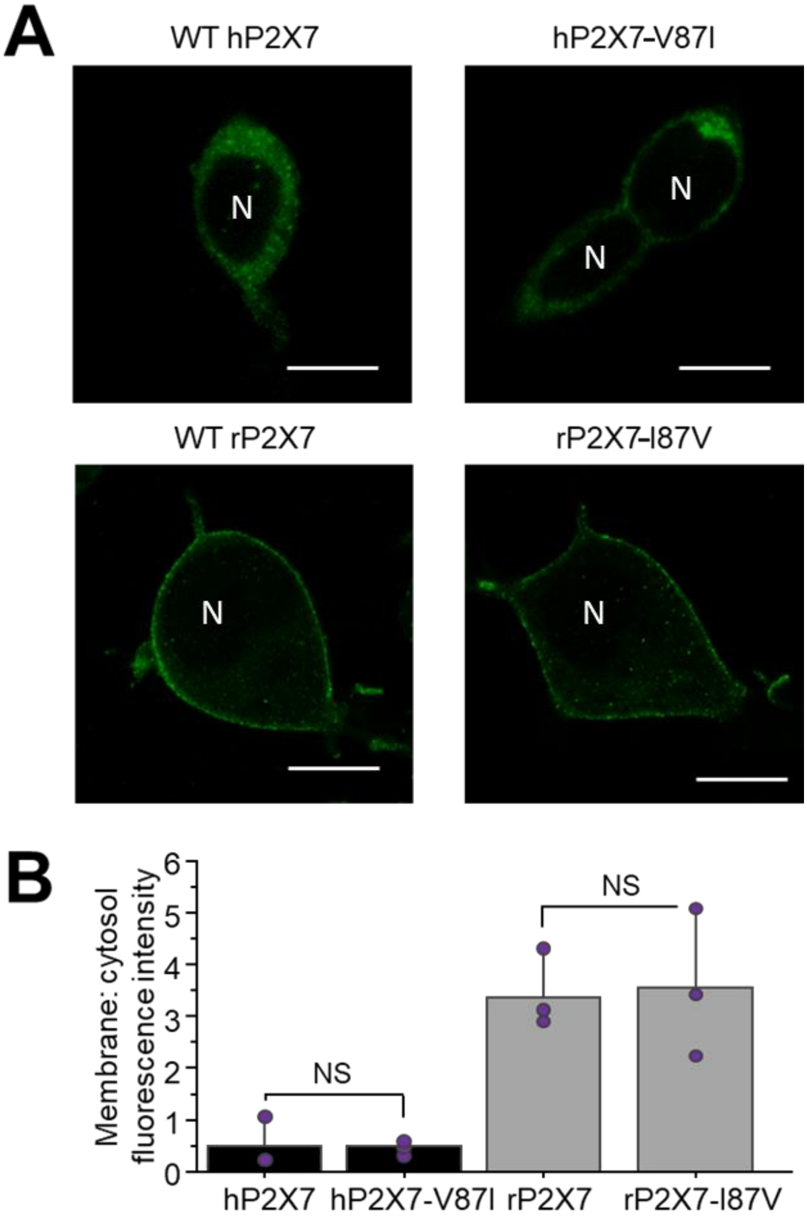

Figure S3 Lack of effect of mutating Val/Ile87 on the expression and subcellular distribution of human and rat P2X7 receptors.
A. Representative confocal images of immunofluorescence show expression and distribution of wild-type (WT) or mutant human and rat P2X7 receptors. N denotes nucleus. The scale bar is 10 μm**. B**. Summary of the membrane/cytosol fluorescence intensity ratio for WT and mutants human and rat P2X7 receptors shown (n = 3 cells in each case).
